# Circularly Polarized X-Band Fan-Beam Antenna and Its Application to Offset Reflector Systems

**DOI:** 10.3390/s26041301

**Published:** 2026-02-17

**Authors:** Tae-Hak Lee, Sang-Gyu Lee, Sang-Burm Ryu, Seongmin Pyo, Ke Wu

**Affiliations:** 1 Department of Electrical and Electronic Engineering, Yuhan University, Bucheon 14780, Republic of Korea; 2 Satellite Payload Research and Development Division, Korea Aerospace Research Institute, Daejeon 34133, Republic of Korea; sglee@kari.re.kr (S.-G.L.); sbryu11@kari.re.kr (S.-B.R.); 3 Department of Information and Communication Engineering, Hanbat National University, Daejeon 34158, Republic of Korea; spyo@edu.hanbat.ac.kr; 4 Poly-Grames Research Center, École Polytechnique, Montréal, QC H3T 1J4, Canada; ke.wu@polymtl.ca

**Keywords:** X-band, circular polarization, microstrip, array, fan-beam, offset reflector, primary pattern

## Abstract

In this letter, a circularly polarized (CP) 4 × 4 array antenna generating a fan-beam radiation pattern is presented, along with its application as the primary pattern of an offset reflector antenna. A sequentially rotated feed network is incorporated into the proposed antenna, enabling a wide axial ratio (AR) bandwidth of 1.9 GHz centered at 8.2 GHz. The proposed array antenna generates about 27.5° and 14.5° of half-power beamwidth (HPBW) in ϕ=0° and ϕ=90° planes, respectively. The fabricated antenna shows good agreement with the simulated results in terms of impedance bandwidth, gain, and radiation characteristics. Furthermore, the offset reflector antenna fed by the proposed CP array is evaluated, resulting in a gain enhancement of approximately 17 dB and a fan-beam radiation characteristic with half-power beamwidths of 3.95° and 2.15°, with an axial ratio bandwidth of 1 GHz.

## 1. Introduction

Circularly polarized (CP) antennas are widely used in advanced communication systems because they effectively reduce polarization mismatch and ensure reliable signal transmission in dynamic environments. Compared with linearly polarized antennas, CP antennas provide improved tolerance to orientation mismatch between transmitting and receiving terminals, which is particularly advantageous in radar systems, global positioning system (GPS), and satellite communication networks [1,2,3]. In addition, the ability of CP antennas to suppress multipath interference contributes to enhanced signal integrity in practical applications such as satellite-based positioning and autonomous navigation systems.

Fan-beam radiation patterns are essential in applications requiring elongated spatial coverage over a specific region [4,5,6,7,8,9]. By providing a wide beamwidth in one plane and a narrow beamwidth in the orthogonal plane, fan-beam antennas enable wide-area observation while maintaining sufficient angular resolution, which is particularly advantageous for remote sensing systems and recent low-Earth orbit (LEO) satellite missions demanding wide swath coverage. Although antennas exhibiting either circular polarization or fan-beam characteristics have been extensively investigated, the integration of both features within a single antenna structure remains challenging. Several approaches have been reported to address this requirement, including CP fan-beam antennas based on electric and magnetic dipole combinations [6], all-metal patch structures supporting dual-frequency CP fan-beam radiation [7], and meta-line-based designs enabling multi-band CP fan-beam operation [8]. Fan-beam characteristics at frequencies above the K-band have also been demonstrated using dual-reflector systems for satellite communication and direct broadcast reception [9]. However, these approaches often involve increased design complexity, fabrication challenges, or limited operational bandwidth. In reflector-based remote sensing systems, the radiation characteristics of the primary feed strongly influence key system-level parameters such as swath width, illumination efficiency, and polarization purity. Fan-beam primary patterns are obviously effective for offset reflector configurations, and when circular polarization is employed, the primary feed can further mitigate polarization distortion caused by reflector geometry and platform motion. Nevertheless, realizing a compact and planar primary feed that simultaneously provides wideband circular polarization and a controlled fan-beam radiation pattern remains challenging [9,10].

In this work, a circularly polarized 4 × 4 array antenna employing a sequentially rotated feed network is proposed to generate a fan-beam radiation pattern. The antenna is based on circular microstrip patch elements arranged asymmetrically to achieve unequal beamwidths in orthogonal planes, while circular polarization is preserved across the array through a sequential phase rotation. In addition, the feasibility of employing the proposed CP array as the primary feed of an offset reflector antenna is investigated, demonstrating its suitability for remote sensing applications requiring wide swath coverage and high antenna gain.

## 2. Antenna Design

**Figure 1:**
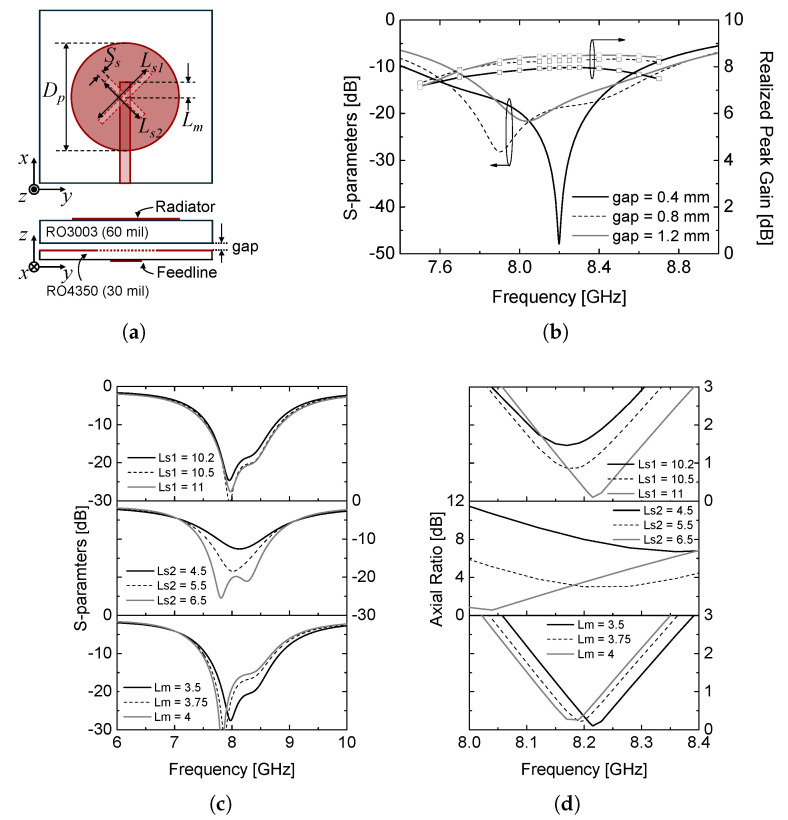
(**a**) Single radiator structure, (**b**) air-gap tuning results, (**c**) impedance matching, and (**d**) axial ratio optimization. ($D_{p}$ = 11.22, $S_{s}$ = 0.80, $L_{s1}$ = 11.00, $L_{s2}$ = 6.25, $L_{m}$ = 3.65, airgap = 0.80, all in [mm]).

The proposed array antenna is designed with a center frequency of an 8.2 GHz for X-band satellite communication systems, targeting an impedance bandwidth greater than 500 MHz. A circular microstrip patch radiator is adopted as the unit radiating element due to its compact geometry and stable radiation characteristics, which are well suited for circularly polarized antenna designs [11,12]. As illustrated in Figure 1a, the fundamental resonant frequencies of two orthogonal modes are primarily determined by the patch diameter, $D_{p}$, while circular polarization is achieved by introducing a cross-shaped coupling slot between the microstrip feed line and the circular patch. The simulated reflection coefficient and axial ratio results, shown in Figure 1b–d, indicate that the impedance matching and CP performance are strongly dependent on the slot dimensions ($L_{s1}$, $L_{s2}$) and the microstrip line length ($L_{m}$). By properly optimizing these parameters, the desired impedance bandwidth and circular polarization performance can be simultaneously achieved. In addition, an air gap between two substrates (RO3003 ($\epsilon_{r}$ = 3.0, tanδ = 0.0013) and RO4350 ($\epsilon_{r}$ = 3.66, tanδ = 0.004)) is introduced. This air gap effectively reduces the effective permittivity of the antenna structure, thereby enhancing impedance matching and improving directivity. The simulation results of the realized peak gain over the operating bandwidth are shown in Figure 1b with different air gap values. The complete set of design parameter values is given in the caption of Figure 1 for repeatability.

**Figure 2:**
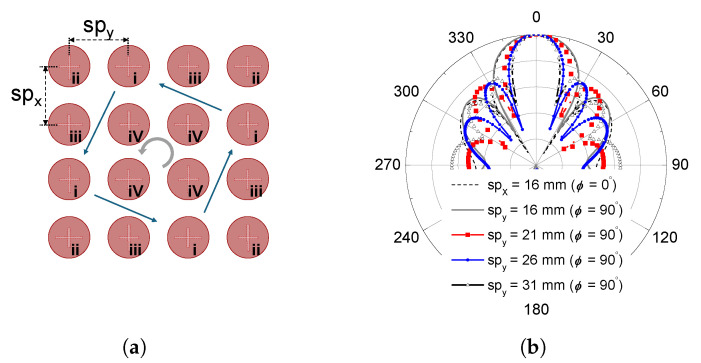
Radiation pattern changes for various inter-radiating patch distances, (**a**) 4 × 4 array structure and (**b**) simulated radiation patterns.

It should be noted that the single radiating element shown in Figure 1 is designed not only to achieve wideband circular polarization but also to ensure stable radiation characteristics suitable for array implementation. By independently optimizing the impedance matching and axial ratio performance at the element level, mutual coupling effects and polarization degradation in the array configuration can be effectively mitigated. Based on this element design, the fan-beam radiation characteristic is primarily realized through the array arrangement, as illustrated in Figure 2, where the inter-element spacing is intentionally controlled to shape the beamwidths in orthogonal planes. This separation of element-level polarization design and array-level beam shaping provides a systematic and flexible approach to achieving the desired fan-beam circularly polarized radiation.

The circular microstrip patch radiators are arranged to form a 4 × 4 array structure, as illustrated in Figure 2a. By adjusting the inter-element spacing along two orthogonal axes, the beamwidths in the corresponding planes can be independently controlled. When the elements are uniformly distributed, identical half-power beamwidths (HPBWs) are obtained in both planes, whereas unequal HPBWs can be realized by introducing asymmetric spacing along a single direction. In the proposed design, the inter-element spacings are chosen as sp_*x*_ = 0.42λ (15.53 mm) and sp_*y*_ = 0.75λ (27.44 mm) at the center frequency of 8.2 GHz, resulting in a fan-beam radiation pattern. The simulated radiation patterns in Figure 2b clearly demonstrate the variation of beamwidth as a function of element spacing. It is worth noting that the inter-element spacing can be further adjusted to meet specific HPBW requirements while considering the position of the first sidelobe. As the HPBW becomes narrower, the first sidelobe shifts closer to the main beam. In remote sensing applications, elevated sidelobe levels or closer sidelobe positions may degrade image quality due to increased ambiguity effects. Therefore, the element spacing in the proposed array is carefully selected to achieve the desired fan-beam characteristic while mitigating potential ambiguity degradation.

**Figure 3:**
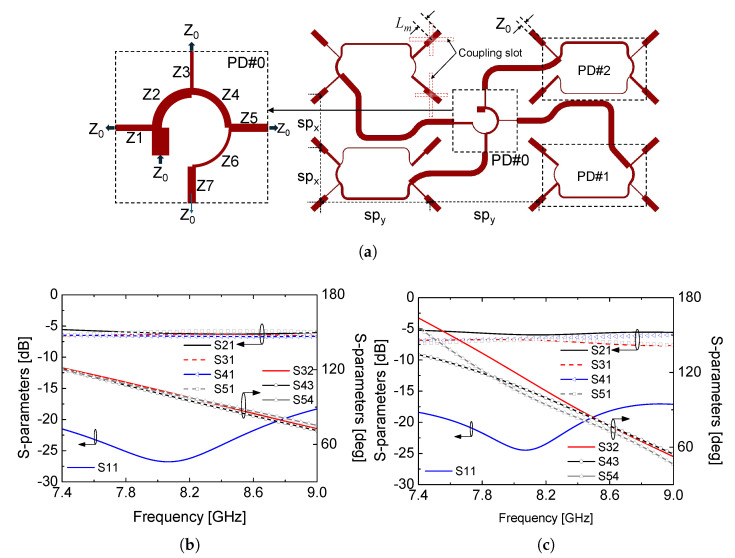
(**a**) Feed line configuration for 4 × 4 radiating elements, (**b**) simulated S-parameters of the PD #0 section, and (**c**) simulated S-parameters of the PD #1 section.

In addition to the asymmetric element placement, the input phase of each radiating element is carefully controlled to realize circular polarization across the array. This is achieved using a microstrip-based sequentially rotated feed network, which is a well-established technique for wideband circular polarization in microstrip patch arrays [13,14,15]. By providing progressive phase shifts to the individual radiating elements while maintaining equal power division, the sequential rotation enables stable CP performance over a wide frequency range. The feed network configuration, illustrated in Figure 3a, consists of a cascade of power dividers (PD #0, PD #1, and PD #2). PD #0 is connected to the 50-Ω input port and distributes the input signal to four subarrays, while PD #1 and PD #2 further divide the signal within each subarray. To satisfy the phase requirements for sequential rotation, additional one-wavelength transmission-line sections are introduced in PD #1 and PD #2. This configuration ensures that the relative phase differences among the output ports remain close to 90°, which is essential for maintaining circular polarization through sequential rotation of the radiating elements. The detailed characteristic impedances and electrical lengths of the feed lines are summarized in Table 1. The simulated S-parameters of the feed network, shown in Figure 3b,c, confirm stable power division and consistent phase progression over the operating frequency band. Although slight differences in phase slope are observed due to unequal line lengths between certain output ports, the impedance matching and power-splitting characteristics are well preserved. As a result, the proposed feed network supports a wide axial ratio bandwidth while preserving the intended fan-beam radiation pattern.

## 3. Experimental Results

**Figure 4:**
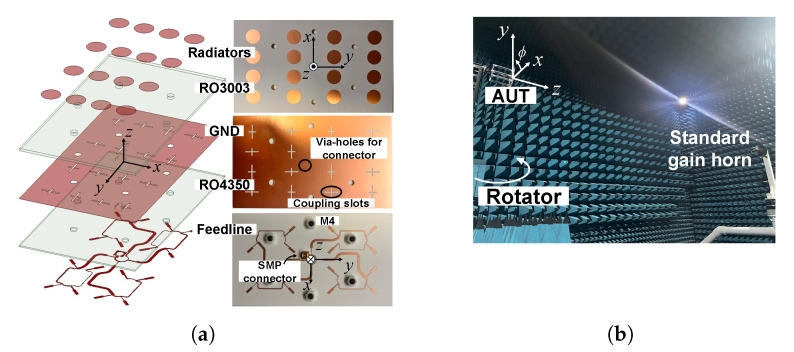
(**a**) Expanded view of the proposed circularly polarized antenna and photographs of the fabricated circuit, (**b**) measurement environment of anechoic chamber (from Korea Electronics Technology Institute, Seongnam-si, Republic of Korea).

In this section, the proposed circularly polarized antenna with fan-beam radiation characteristics is experimentally validated through measurements. Figure 4a shows photographs of the fabricated antenna, including the radiating elements and the integrated feed network, while the measurement environment inside an anechoic chamber is illustrated in Figure 4b. The antenna under test is mounted on a rotator aligned with the defined coordinate system to accurately capture the radiation characteristics in the orthogonal planes. A 50-Ω SMP connector (SMP-MSLD-20T) is employed at the input port to ensure reliable electrical connection and repeatable measurement conditions over the X-band frequency range.

**Figure 5:**
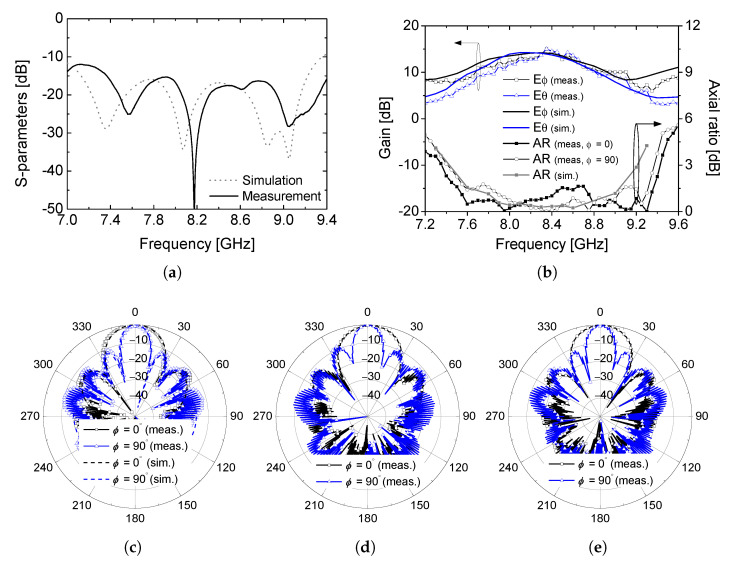
Simulation and measurement comparison, (**a**) S_11_, (**b**) gain and axial ratio, (**c**) radiation patterns at 8.2 GHz, (**d**) at 8.0 GHz, and (**e**) at 8.4 GHz.

The measured reflection coefficient of the fabricated antenna is compared with the simulated result in Figure 5a. Good agreement is observed between simulation and measurement, with the impedance bandwidth centered at 8.2 GHz and exceeding 500 MHz, which satisfies the design requirement for X-band satellite communication systems. Minor discrepancies between the measured and simulated results are mainly attributed to the non-ideal parameters such as fabrication tolerances and connector effects, which are not fully accounted for in the simulation process.

Figure 5b presents the measured gain and axial ratio (AR) performances of the proposed array antenna. The measured gain remains stable across the operating frequency band and closely follows the simulated trend, confirming the effectiveness of the sequentially rotated feed network in maintaining uniform power distribution and phase progression. The axial ratio remains below 3 dB from 7.45 GHz to 9.35 GHz in both the ϕ = 0° and 90° planes, demonstrating that the circular polarization performance of the single element is well preserved in the array configuration despite mutual coupling effects. The measured radiation patterns at 8.0 GHz, 8.2 GHz, and 8.4 GHz are shown in Figure 5c–e and compared with the simulated results. In all cases, the measured patterns exhibit good agreement with simulations in both principal planes. At the center frequency of 8.2 GHz, the antenna clearly demonstrates the intended fan-beam radiation characteristic, with unequal half-power beamwidths (HPBWs) of approximately 28° and 12° in the orthogonal planes. These values are consistent with the simulated HPBWs of 27.5° and 14.5°, confirming that the asymmetric inter-element spacing effectively controls the beam shaping while maintaining circular polarization. Furthermore, the fan-beam radiation characteristic is consistently maintained across the operating frequency band, indicating that the proposed array configuration provides stable beam shaping without significant pattern distortion.

A comparison with previously reported circularly polarized and fan-beam antenna designs is summarized in Table 2. The proposed antenna achieves a significantly wider impedance bandwidth of 6.0–9.6 GHz (43.9%) and a 3-dB axial ratio bandwidth of 7.45–9.35 GHz (23.17%), which are notably broader than those of many previously reported CP fan-beam antennas. In addition, the antenna provides fan-beam characteristics with half-power beamwidths of approximately 28° and 12° while maintaining a competitive peak gain of 17.1 dB. These results are achieved using a compact and fully planar structure, highlighting the effectiveness of the proposed design in simultaneously realizing wideband circular polarization, stable fan-beam radiation, and practical integration capability. Such features demonstrate the suitability of the proposed array as a primary feed candidate for X-band reflector-based satellite communication or remote sensing applications.

## 4. Application to Offset Reflector Systems

**Figure 6:**
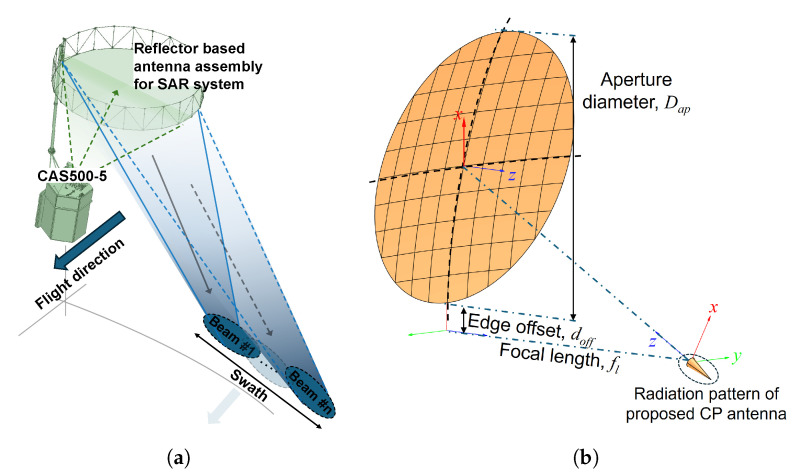
(**a**) Fan-beam application for CAS500-5 LEO satellite, (**b**) offset reflector optical configuration for simulation.

For low-earth orbit (LEO) satellite remote sensing missions, wide swath coverage is a key system requirement, which is commonly achieved using reflector-based antenna architectures with asymmetric radiation characteristics. Offset reflector configurations are particularly attractive in such systems because they reduce feed blockage and allow flexible control of illumination distribution across the reflector aperture. Compared with conventional horn feeds, the proposed CP array provides a compact and planar alternative that can simultaneously generate circular polarization and a fan-beam radiation pattern without additional shaping structures. This allows effective control of the illumination distribution along the swath direction and enables evaluation of how the axial ratio and gain characteristics of the reflector system vary depending on the primary feed performance. As illustrated in Figure 6a, fan-beam radiation patterns are often employed to extend the observation swath along the flight direction, which is essential for multi-beam synthetic aperture radar (SAR) payloads such as the CAS500-5 mission [16]. To evaluate the feasibility of the proposed circularly polarized array antenna as a primary feed, its radiation characteristics are applied to an offset reflector antenna model, whose optical parameters are shown in Figure 6b. In this study, the simulated far-field patterns of the proposed array antenna are exported and used as the excitation source in a TICRA GRASP analysis, allowing accurate assessment of the combined antenna performance including polarization effects [17]. The reflector geometry is designed to provide high gain while preserving the polarization characteristics of the primary feed. The aperture diameter, $D_{\mathrm{ap}}$ is determined to be 50 cm with a 0.8 of focal length to diameter ratio ($f_{l}$/$D_{\mathrm{ap}}$) and the edge offset, $d_{\mathrm{off}}$ is designed to be 15 cm. The circular polarization performance of the reflector antenna is evaluated using the axial ratio definition given by (1), which is derived from the right-hand and left-hand circularly polarized field components. This formulation enables direct comparison between the polarization characteristics of the primary feed and those of the reflector antenna, providing insight into polarization preservation through the reflection process.

(1) $$AR_{\mathrm{dB}}=20\log\left(\frac{|E_{RH}\left|+\right|E_{LH}|}{\left|\right|E_{RH}\left|-\right|E_{LH}\left|\right|}\right)$$

**Figure 7:**
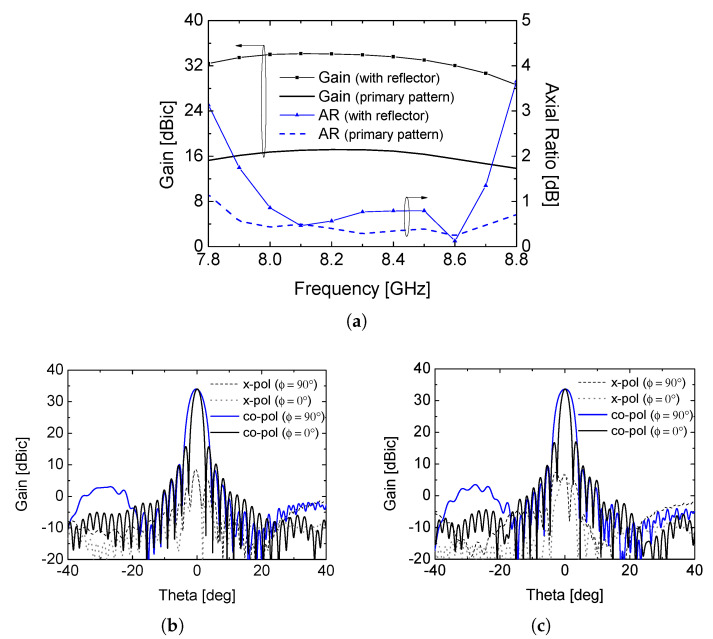
(**a**) comparison of simulated gain and AR, (**b**) radiation pattern at 8.0 GHz, and (**c**) at 8.4 GHz. ($D_{\mathrm{ap}}$ = 1 m, $l_{f}$ = 1.2 m, $d_{\mathrm{off}}$ = 0.1 m).

Figure 7a–c present the simulated gain and radiation characteristics of the offset reflector antenna fed by the proposed CP array. Over the frequency range from 8.0 GHz to 8.4 GHz, the reflector antenna achieves a gain enhancement of approximately 17 dB compared to the primary feed, resulting in a peak gain of 33.5 dB. At the same time, the 3-dB axial ratio bandwidth is maintained from 7.8 GHz to 8.8 GHz, indicating that the circular polarization performance of the primary feed is effectively preserved after reflection.

The resulting secondary radiation patterns exhibit fan-beam characteristics with half-power beamwidths of approximately 4.0° and 2.0° in the ϕ = 90° and 0° planes, respectively. These asymmetric beamwidths are consistent with the illumination provided by the primary feed and demonstrate that the proposed array antenna is well suited for reflector-based wide-swath remote sensing systems. Furthermore, the cross-polarization levels remain sufficiently low across the operating frequency band, confirming the robustness of the proposed CP feed when integrated with an offset reflector.

## 5. Conclusions

A circularly polarized 4 × 4 array antenna with a sequentially rotated feed network has been presented to realize a fan-beam radiation pattern. The proposed array achieves unequal half-power beamwidths of approximately 28° and 12° in orthogonal planes while maintaining wide axial ratio bandwidth from 7.45 GHz to 9.35 GHz, as verified by measurements. In addition, the feasibility of employing the proposed CP array antenna as the primary feed of an offset reflector has been examined, confirming its applicability to remote sensing systems requiring circular polarization and wide swath coverage.

## Figures and Tables

**Table 1:** Feed line design parameters.

	Z_0_	Z_1_	Z_2_	Z_3_	Z_4_	Z_5_	Z_6_	Z_7_
Ω	50	100	80	120	96	80	128	80
λ	PD0	$\lambda_{g}$/4						
	PD1	$\lambda_{g}$/4			$5\lambda_{g}/4$	$\lambda_{g}$/4		
	PD2	$\lambda_{g}$/4	5$\lambda_{g}$/4	$\lambda_{g}$/4			5$\lambda_{g}$/4	$\lambda_{g}$/4

**Table 2:** Comparison table.

Radiation Elements	Size [$\lambda_{0}^{3}$]	10 dB S_11_ Band- Width [GHz]	3 dB AR Band- Width [GHz]	Hpbw, Peak Gain at *f*_0_
[3] Loop	0.46 × 0.06 × 0.004	2.27–2.57 (10.0%)	2.482–2.501 (0.76%)	109°/84°, 4.80 dBic
[4] 1 × 4	4.59 × 0.71 × 0.08	3.35–3.75 (11.27%)	linear polarization	85.44°/23.86°, 10.91 dB
[5] 1 × 4	2.26 × 0.63 × 0.2	4.52–5.83 (22.98%)	linear polarization	89.13°/20.72°, 9.05 dB
		9.08–9.66 (5.98%)		82.44°/12.81°, 11.3 dB
[6] Patch	0.60 × 0.32 × 0.029	5.7–5.9 (3.5%)	5.65–5.90 (4.3%)	168°/85°, ∼3.75 dB
[7] 1 × 8	6.21 × 2.07 × 0.65 (given vol.)	7.0–9.0 (25%)	7.145–7.190 8.40–8.45 (requirements)	140° (3 dB)/10°, 16.1 dBic
				140° (5 dB)/ 10°, 17.5 dBic
[8] Metaline	1.31 × 0.44 × 0.028	2.0–4.0 (66.6%)	10.4% of 2.6 GHz	102°/44°, 7.0 dB
			9.6% of 3.6 GHz	96°/45°, 6.9 dB
[9] 2 × 2, DR	reflector system	11.7–12.0, (2.53%) 20.355–21.155 (3.85%) 30.085–30.885 (1.31%)		3.3°/1.0°, 4.0°/1.3°, 6.4°/2.6°, 39.6, 36.5, 29.3 dBi
this work 4 × 4	3.42 × 1.97 × 0.084	6.0–9.6 (43.9%)	7.45–9.35 (23.17%)	28°/12°, 17.1 dB

## Data Availability

The original contributions presented in this study are included in the article. Further inquiries can be directed to the corresponding author.
